# Psychometric Properties of a New Measure of Upper Limb Performance in Post-Stroke Individuals: Trunk-Based Index of Performance

**DOI:** 10.1177/15459683221143462

**Published:** 2022-12-28

**Authors:** Daniele Piscitelli, Melanie C. Baniña, Timothy K. Lam, Joyce L. Chen, Mindy F. Levin

**Affiliations:** 1 School of Physical and Occupational Therapy, McGill University, Montreal, QC, Canada; 2 Feil/Oberfeld Research Centre of the Jewish Rehabilitation Hospital/Centre for Interdisciplinary Research in Rehabilitation, Laval, QC, Canada; 3 Department of Kinesiology, University of Connecticut, Storrs, CT, USA; 4 Canadian Partnership for Stroke Recovery, Hurvitz Brain Sciences Research Program, Sunnybrook Research Institute, Toronto, ON, Canada; 5 Faculty of Kinesiology and Physical Education, University of Toronto, Toronto, ON, Canada

**Keywords:** stroke, upper limb, assessment, neurological examination, psychometrics, kinematics, recovery

## Abstract

**Background:**

Several measures of upper limb (UL) motor tasks have been developed to characterize recovery. However, UL performance and movement quality measures in isolation may not provide a true profile of functional recovery.

**Objective:**

To investigate the measurement properties of a new trunk-based Index of Performance (IPt) of the UL combining endpoint performance (accuracy and speed) and movement quality (trunk displacement) in stroke.

**Methods:**

Participants with stroke (n = 25, mean time since stroke: 18.7 ± 17.2 months) performed a reaching task over 3 evaluation sessions. The IPt was computed based on Fitts’ Law that incorporated endpoint accuracy and speed corrected by the amount of trunk displacement. Test–retest reliability was analyzed using intraclass correlation coefficient (ICC) and Bland–Altman plots. Standard error of measurement (SEM) and Minimal Detectable Change (MDC) were determined. Validity was investigated through the relationship between IPt, Fugl–Meyer Assessment (FMA-UE), and Action Research Arm Test (ARAT), as well as the ability of IPt to distinguish between levels of UL motor impairment severity.

**Results:**

Test–retest reliability was excellent (ICC = .908, 95% CI: 0.807-0.96). Bland–Altman did not show systematic differences. SEM and MDC_95_ were 14% and 39%, respectively. Construct validity was satisfactory. The IPt showed low-to-moderate relationships with FMA-UE (*R*^2^ ranged from .236 to .428) and ARAT (*R*^2^ ranged from .277 to .306). IPt scores distinguished between different levels of UL severity.

**Conclusions:**

The IPt showed evidence of good reliability, and initial validity. The IPt may be a promising tool for research and clinical settings. Further research is warranted to investigate its validity with additional comparator instruments.

## Introduction

The first consensus meeting of the Stroke Recovery and Rehabilitation Roundtable (SRRR) concluded that more accurate and predictive kinematic measures of upper limb (UL) function need to be developed to distinguish between changes in behavior due to functional recovery or compensation.^1^ They concluded that more informative measures of motor behavior are vital to the understanding of neural repair processes and training effects on motor action.^2,3^ Consequently, the second SRRR consensus panel recommended 4 performance “assays” to characterize real change at the body structure/function level and 1 functional task at the activity level.^4^ In this context, “assay” refers to a movement component that is applicable to a wide range of motor actions or tasks. Assays were chosen based on their ability to quantify the execution of a movement component outside the context of the performance of any specific motor task. They include a measure of coordination between shoulder and elbow movements; finger force individuation; maximal isometric grip and pinch strength. At the activity level, evaluation of endpoint kinematics (eg, speed, smoothness), and joint and trunk displacements during a standardized reaching task were recommended.^4^

Although deviations from typical movement patterns provide information about the motor elements contributing to task performance, it is unclear how individual elements, including the presence of motor compensations, reflect skilled movement. Regarding UL reaching, skilled movement has been defined as an action that is executed at a short latency with high speed and precision.^5^ This is often referred to as the speed–accuracy trade-off relationship, based on the concept of Fitts’ law.^6^ Fitts’ Law results in a measure that integrates the time to move the hand to the target as a function of the hand-to-target distance and the target width, but does not account for the influence of compensatory trunk movements. Quantification of compensatory movement is vital to the development of a metric for skilled reaching since, in contrast to healthy participants, in patients with moderate-to-severe stroke, endpoint precision is often influenced by trunk displacement.^7,8^

We propose a novel application of Fitts’ Law characterizing skilled reaching in people with stroke, that also accounts for the amount of trunk displacement used during reaching, called the Trunk-based Index of Performance, IPt. The new measure incorporates trunk displacement in the classic Index of Performance (IP) measure, which is expressed as the Index of Difficulty (ID) of the task divided by the Movement Time (MT; IP = ID/MT), where ID is a function of the reaching distance (*D*) to the target divided by the target width (*W*), such that ID = log_2_ (2*D*/*W*).

The classic IP has been used in numerous experiments in motor control, psychology, and neuroscience.^9,10^ However, it has received less attention in studies of the recovery of reaching skill in the neurorehabilitation literature^11^ (but see Smits-Engelsman et al^12^), in which motor skill is usually characterized as smaller endpoint error and/or faster movement speed.^13^ The new measure, IPt, may be better able to capture real-world manifestations of motor skill learning, where increased skill is inferred if both variables change in the expected direction (faster speed, increased precision),^14^ with less trunk compensation.

The study goal was to determine the clinimetric properties, that is, reliability (ie, test–retest reliability, measurement error) and validity of IPt as a measure of motor skill in people with chronic stroke performing a standardized reaching task. We hypothesized that IPt would show high test–retest reliability and discriminate between participants with mild or moderate-to-severe hemiparesis. Preliminary results have appeared in abstract form.^15^

## Methods

Data used for this study were collected as part of a double-blind, crossover randomized controlled trial (https://www.clinicaltrials.gov, NCT02473549). Participants underwent 3 sessions of reaching training in which they received either anodal, cathodal, or sham Transcranial Direct Current Stimulation (tDCS), separated by 2 week periods. Before each training session, pre-test arm functional ability was assessed with standard clinical scales and a standardized reaching Test Task (see below). Kinematic data from the 3 pre-test Test-Tasks were used for analysis (ie, session-1, session-2, session-3).

### Participants

Individuals with stroke were recruited from rehabilitation hospitals in Toronto (Ontario) and Laval (Quebec), Canada. Inclusion criteria were: unilateral first-time ischemic stroke in middle cerebral artery territory; stroke onset between 3 months and 5 years previously; age >18 years; mild to severe motor deficits (Chedoke-McMaster Arm Impairment Inventory (CMSA ARM ≥3)^16,17^; able to perform ~30° of active elbow extension; English or French speaking. Exclusion criteria were: moderate-to-severe spasticity (Modified Ashworth Scale (MAS) >3^18^; significant cognitive deficits (<23/30, Montreal Cognitive Assessment^19,20^); severe apraxia (<2 SD below the healthy control mean; Waterloo Apraxia test^21,22^); neglect (>40/100, Sunnybrook Neglect Assessment Procedure)^23^; previous musculoskeletal problems in UL and/or back; neurodegenerative or psychiatric disease; contraindications to magnetic resonance imaging and tDCS; and taking medications known to modulate tDCS effects. The study was approved by Research Ethics Boards at Sunnybrook Health Sciences Centre (Toronto) and the Montreal University Health Centre (Montreal). All participants provided written informed consent.

### Clinical Assessments

Three licensed health-care professionals (1 occupational and 2 physical therapists) assessed the clinical status of participants using valid assessments. Handedness was determined with the Edinburgh Handedness Inventory^24^ in which participants indicate their hand preference for 10 UL activities. Arm impairment was assessed with the Fugl–Meyer Assessment of the Upper Extremity (FMA-UE) and activity limitation was assessed with the Action Research Arm Test (ARAT). FMA-UE^25,26^ assessed voluntary movement, coordination, and reflex activity. Each item was scored on a 3pt ordinal scale; 0 (cannot be performed), 1 (partially performed), and 2 (performed entirely) for a maximum score of 66pt. ARAT^27^ assessed the ability to handle objects differing in size, weight, and shape on 4-pt ordinal scales ranging from 0 (unable to complete any hand/arm movements) to 3 (completes in <5 seconds)^28^ for a maximum score of 57pt.

### Reaching Test Task

**Figure 1.**
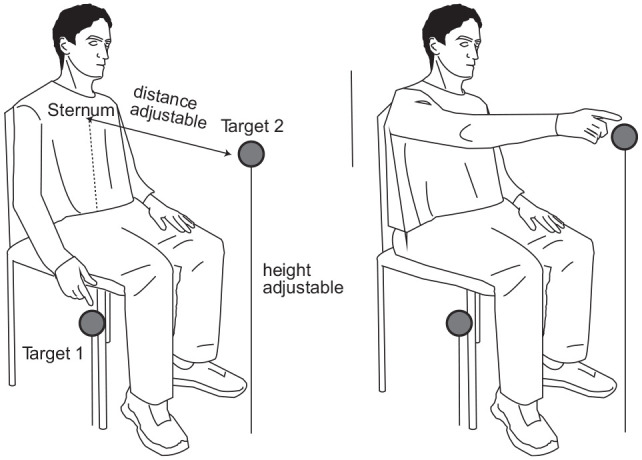
Experimental set-up for the Test Task.

A standardized reaching task representing a common motor task of activities of daily living,^29^ was used to measure kinematics (Reaching Test Task,^30^ Figure 1).

Participants were seated in an armless chair with their back supported but unrestrained. In the initial position, the hemiparetic arm was alongside the body with the hand placed on a platform at the ipsilateral greater trochanter level. In this position, the arm position was 0° shoulder flexion, 40° elbow flexion, 20° wrist extension and the hand was in neutral.

Participants were instructed to reach-to-touch a 6 cm × 6 cm target as precisely and as quickly as possible with the third metacarpal (closed-hand position; 25 trials) with eyes open. The closed-hand position ensured that participants without sufficient finger control could perform the task. The target was located in the trunk midline at sternal notch height and at arm’s length, measured from the medial mid-axillary border to the third metacarpal fingertip, with the elbow extended. Participants had 5 practice trials prior to recording for task familiarization.

### Kinematic Data Acquisition and Analysis

UL and trunk kinematics were recorded with an Optotrak motion capture system (Northern Digital, Canada, 100 Hz) with 6 markers placed on the endpoint (third metacarpal head), wrist (ulnar head), elbow (lateral epicondyle), shoulders (ipsilateral and contralateral acromions), trunk (mid-sternum), and one marker on the target (Figure 1). Note that for the IPt computation, only markers on the third metacarpal head, sternum, and target were used.

Reaching was characterized by endpoint and joint kinematics. For all measures, movement onset/offset were determined from the endpoint (*x*, *y*, *z*) marker and defined as the times when the tangential velocity trace rose/fell and remained above/below 10% of the mean peak velocity of the trial for a minimum of 25 ms. For the endpoint, trajectory length was defined as the real distance (mm) from initial to final position, incorporating *x*, *y*, and *z* displacements of the endpoint marker. MT was defined as the duration (ms) of the reach from movement onset-to-offset.

Trunk displacement was measured as the sagittal distance (mm) moved by the sternal marker between movement onset and offset, where positive values indicated forward displacement from the initial position (0 mm). Data were interpolated (third-order spline function) and low-pass filtered at 20 Hz. Data analysis was performed with custom-made programs (Matlab 2018a, MathWorks, Natick, MA).

### Trunk-Based Index of Performance

The IPt was computed for each trial of the Reaching Test Task and then averaged over all trials for each subject and session separately. Equation (1) defines the IPt computation:

(1) $$\mathrm{IPt}=\frac{IDe}{MT}$$

where MT is the movement time, and IDe is the effective ID (equation (2)).

(2) $$\mathrm{IDe}=\log_{2}\left(\left(\frac{\sum_{i=1}^{N}\left(D-t\right)}{N}÷We\right)+1\right)$$

where *D* represents the sagittal distance moved by the endpoint from onset-to-offset and *t* represents the sagittal trunk displacement for each trial. N is the number of trials per session. “We” represents the effective width (as defined in Fitt’s law^6^) of the final target (defined for each participant), computed as the standard deviation of the total error of all trials multiplied by the 95% CI *z*-value, that is, We = 1.96 × SD_TotalError. TotalError represents the *x*, *y*, *z* distance in mm of the endpoint marker from the target position based on the root mean square error, where RMSE = $\sqrt{x^{2}+y^{2}+z^{2}}.$ Note that by subtracting the amount of sagittal trunk displacement from the endpoint movement, equation (2) accounts for the effect of the trunk motion on the reaching task.^31^

### Statistical Analysis

#### Reliability

Reliability, which evaluates the extent to which a measurement is free from measurement error,^32^ *was investigated in terms of* test–retest reliability and measurement error. Test–retest reliability indicates the degree to which the measure is stable over time. The 2-week time interval of observations was considered appropriate for expecting no changes in reaching task performance and clinical outcomes. Participants received no additional treatment during the trial period, and thus, no clinical fluctuations were expected. Variance component analysis was used to evaluate test–retest reliability, that is, to determine if IPt data measured at 3 different time points were stable. A one-way repeated measures ANOVA was performed to investigate the presence of systematic bias within the 3 evaluations. Mauchly’s tests were performed to check sphericity assumptions of data. Greenhouse-Geisser DF adjustments were applied for non-spherical data. The test–retest reliability of the IPt was evaluated with the Intraclass Correlation Coefficient (ICC_2,k_ 2-way random-effects model)^33^ with its 95% confidence intervals (95% CI). ICC values >.90 were considered excellent, while values in the range .75 to .90 and <.75 were considered good and poor-to-moderate, respectively.^34^

Further examination of reliability was conducted through Bland–Altman plot analysis^35^ which evaluates the presence of intra-subject variations and systematic measurement differences, that is, monotonic drift and systematic error increase. Bland–Altman plots the difference between the measurements (eg, session-1 scores minus session-2 scores) against the mean of the same measurements. The 95% limits calculated as the mean difference ± 1.96SD of agreement (LoA_95_) were reported. Thus, 95% of the differences between the 2 measurements are expected to lie between these limits. LoA_95_ values can be approximated to the threshold of clinical differences.

Measurement error corresponds to the change in the measure due to random and systematic error unrelated to true changes in the constructs measured by the instrument (ie, motor quality and motor performance). Measurement error was evaluated by the Standard Error of Measurement (SEM) and the Minimal Detectable Change (MDC). SEM was computing as: SEM = SDpooled × √(1−ICC),^36^ where SD corresponds to the standard deviation of session-1 values. SEM represents the precision of a single score within subjects, also referred to as absolute reliability.^37^

MDC95 was computed by multiplying the SEM by the *z*-score associated with the 95% CI and the square root of 2 (ie, MDC95 = SEM × 1.96 × √2).^34^ MDC identifies the margin of error for detecting a true change in the measured construct. Further, SEM and MDC95 estimates were divided by the mean IPt scores and multiplied by 100 to calculate percentage values independent of measurement units.^38^

### Validity

Validity evaluates if an instrument measures the intended construct.^32^ Construct validity was investigated through a-priori hypotheses testing^32^ of the expected relationship between IPt scores and clinical outcomes (ie, FMA-UE and ARAT). FMA-UE and ARAT were chosen as comparator instruments, because their psychometric properties in the stroke population are well-established and they represent the most commonly used constructs for UL motor function and activity in stroke. A low-to-moderate relationship was expected because the IPt is considered a task-specific kinematic measure incorporating endpoint accuracy and speed (ie, motor performance) and the amount of sagittal trunk displacement (ie, movement quality). In contrast, FMA-UE and ARAT measure UL motor impairment and activity, respectively. Separate regressions were run for studying the relationships. The *R*^2^ was reported as a measure of the amount of variance of the dependent variable (ie, IPt) explained by the independent variables (FMA-UE and ARAT).

Furthermore, we expected a significant ability of IPt to discriminate between subjects with mild or moderate-to-severe hemiparesis. The FMA-UE cutoff score of <50 indicated a moderate–severe impairment level.^39^ The Receiver Operating Characteristic (ROC) curve was plotted and the area under the curve (AUC) computed. An AUC >0.7 was considered adequate.^40^ The ROC curve and Youden index with the 95% CI after bootstrapping (1000 replicates and 900 random-number seeds) were computed using MedCalc 14.10.2 software (MedCalc, Ostend, Belgium). For statistical analyses, the α value was set at *P* < .05. SPSS software was used, Version 17.0 (SPSS Inc., Chicago, IL, USA).

## Results

### Participants

Data from 25 participants (aged 60.6 ± 11.7 years) with stroke (18.7 ± 17.2 months) were available to analyze the reaching task (Table 1).

**Table 1.** Demographic and Clinical Characteristics of Study Participants.

Characteristics	n = 25
Age, years, mean (±SD)	60.6 (±11.7)
Sex (male/female)	22/3
Side of stroke, no. (%)	
Left	40%
Right	36%
Unknown	24%
Handedness, no. (%)	
Ambidextrous	4%
Left	8%
Right	84%
Time since stroke, mean (±SD), months	18.7 (±17.2)
Stroke type no. (%)	
Ischemic	52%
Hemorrhagic	16%
Not recorded	32%
Clinical and functional status	
CMSA-A, median [IQR]	5 [1]
CMSA-H, median [IQR]	4 [2]
MoCA score, median [IQR]	26 [4]
FM-UE score, mean (±SD)	44.7 (±12.9)
ARAT score, mean (±SD)	31.4 (±19.3)
MAS elbow flexors (±SD)	1.3 (±0.9)
MAS wrist flexors (±SD)	1.2 (±0.8)

Abbreviations: CMSA-A, Chedoke-McMaster Stroke Assessment-Arm; CMSA-H, Chedoke-McMaster Stroke Assessment-Hand; MoCA, Montreal Cognitive Assessment; FMA-UE, Fugl–Meyer Assessment Upper Limb; ARAT, Action Research Arm Test; MAS, Modified Ashworth Scale; IQR, interquartile range; SD, standard deviation.

### Reaching Task

The task was well-tolerated with no subject reporting fatigue or discomfort across the 3 sessions. All data met assumptions of linearity, normality, and homogeneity. The mean number of reaching trials across subjects was similar for the 3 evaluations (session-1: 21.9 ± 2.8; session-2: 23.1 ± 3.2; session-3: 22.2 ± 2.4). Some trials were not considered due to failures of kinematic data collection (eg, missing markers, recording started after movement onset).

### Reliability

No significant differences were found among IPt values across the 3 sessions (ie, session-1: 4.51 ± 2.62; session-2: 4.72 ± 2.03; session-3: 4.95 ± 1.93; *F*_(2,38)_ = 0.856, *P* = .433), where higher values indicate better performance. An excellent test–retest reliability was demonstrated by an ICC of .908 (95% CI: 0.807, 0.96; *F*_(19,38)_ = 0.856, *P* < .001). The SEM was 0.671 and the MDC95 was 1.90. The SEM% was 14%, while the MDC95 was 39%.

**Figure 2.**
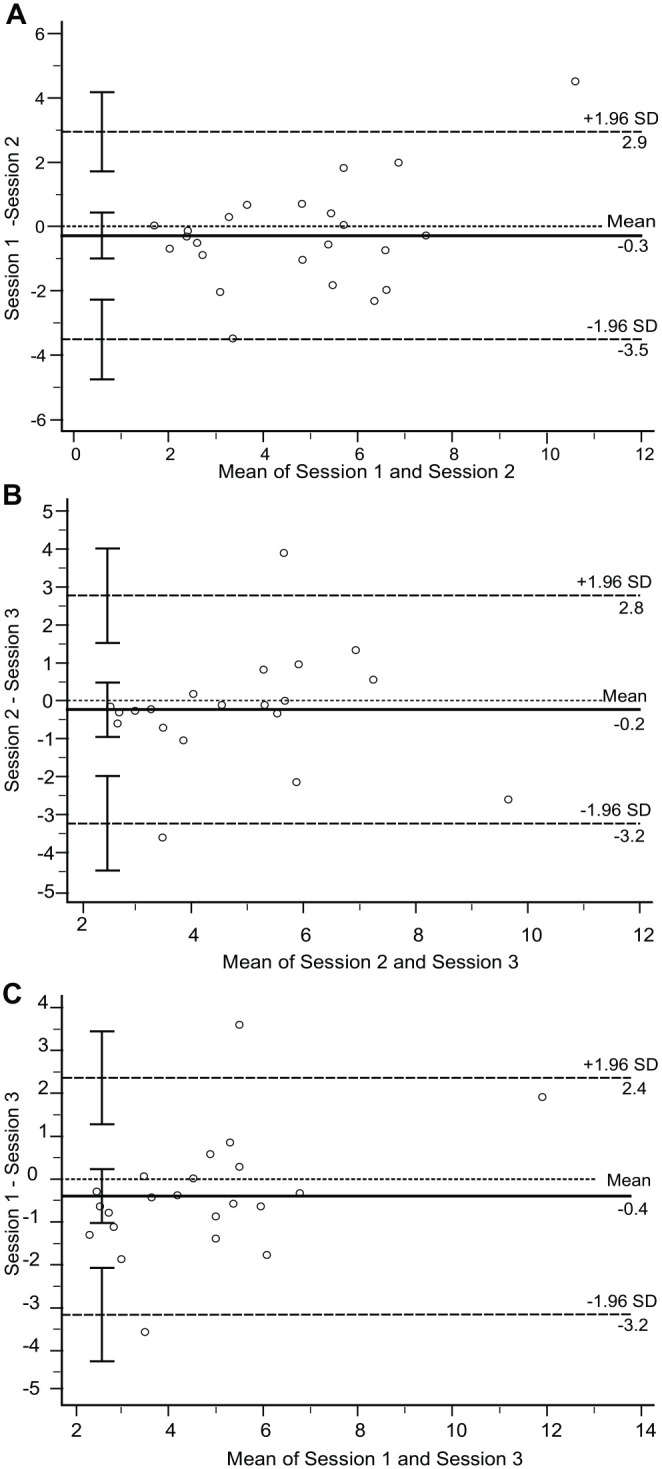
Bland–Altman (BA) plots for trunk-based Index of Performance (IPt) tests. The following pairs were analyzed: (A) IPt session-1 versus IPt session-2; (B) IPt session-2 versus IPt session-3; (C) IPt session-1 versus IPt-session 3. For the 3 BA plots, the mean of paired results is illustrated on the *x*-axis, and the difference between the paired results is depicted on the *y*-axis. The middle horizontal line indicates the mean differences. The 2 horizontal dashed lines indicate the 95% upper and lower limits of agreement. The vertical bars represent the 95% confidence interval for each estimate.

The Bland–Altman plots (Figure 2) depict the difference in the IPt values against the mean IPt values across 3 combinations, that is, session-1 versus session-2, session-3 versus session-2, and session-1 versus session-3. For all 3 plots, the mean difference approached 0, indicating the absence of systematic bias. Two outliers were shown outside the 95% CI limit of agreement.

### Validity

Multiple regressions were performed to investigate the relationship between IPt and clinical outcome measures. The *R*^2^ across the 3 sessions ranged from low-to-moderate for both FMA-UE and ARAT. For FMA-UE, session-1 had a *R*^2^ = .236 (*F*_(1,23)_ = 7.124, *P* = .014; β = 2.487, *t* = 2.669, *P* = .014), session-2 had a *R*^2^ = .428 (*F*_(1,21)_ = 15.694, *P* = .001; β = 3.891, *t* = 3.962, *P* = .001) and session-3 had a *R*^2^ = .284 (*F*_(1,20)_ = 7.934, *P* = .011; β = 3.822, *t* = 2.817, *P* = .011). Similar results were found with ARAT, that is, session-1: *R*^2^ = .306 (*F*_(1,22)_ = 9.691, *P* = .005; β = 4.128, *t* = 3.113, *P* = .005); session-2: *R*^2^ = .313 (*F*_(1,20)_ = 9.121, *P* = .007; β = 5.452, *t* = 3.020, *P* = .007) and session-3: *R*^2^ = .277 (*F*_(1,19)_ = 7.278, *P* = .014; β = 5.467, *t* = 2.698, *P* = .014).

**Figure 3.**
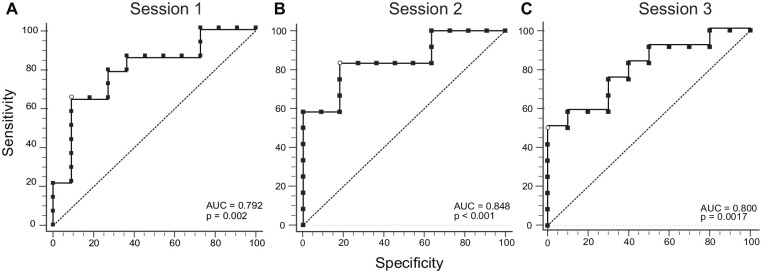
Receiver-Operating-Characteristic (ROC) curves for the trunk-based Index of Performance (IPt) across the 3 sessions. (A) ROC for session-1. (B) ROC for session-2. (C) ROC for session-3. The dashed line represents the reference line for the null hypothesis of the Area Under the Curve (AUC) of 0.5. The open circles show the criterion value based on the Youden index.

The IPt ROCs showed significant AUCs across the 3 sessions (Figure 3). The AUC for session-1 was 0.792 (95% Bootstrap CI: 0.546, 0.942, *P* = .0022), with a Youden index of *J* = 0.5519 (associated criterion = ≤3.520, 95% Bootstrap CI: ≤1.677, ≤5.180), the sensitivity was 64.29% and the specificity was 90.91%. The ROC curve for session-2 yielded an AUC of 0.848 (95% Bootstrap CI: 0.584, 0.955, *P* < .0001). The sensitivity of 83.33% and specificity of 81.82% were associated with an Youden index *J* of 0.6515 (associated criterion = ≤5.106, 95% Bootstrap CI: ≤3.172, ≤6.965). Session-3 showed an AUC of 0.800 (95% Bootstrap CI: 0.569, 0.950, *P* = .0017), an Youden index *J* = 0.50 (associated criterion = ≤3.458, 95% Bootstrap CI: ≤2.986-≤4.600) with a sensitivity of 50% and a specificity of 100%.

## Discussion

According to SRRR recommendations, there is a growing interest in providing researchers and clinicians with valid and meaningful UL kinematic measures to characterize sensorimotor improvements in post-stroke survivors.^1^ A measure that combines movement performance and quality is highly desirable to help distinguish between restitution (ie, re-appearance of premorbid movement patterns) and compensation strategies (ie, recruitment of alternate joint combinations, such as trunk displacement).^41^ The IPt is a novel kinematic measure based on Fitts’ Law to evaluate movement performance and quality in a reaching task in stroke. Use of IPt in research and clinical applications first requires knowledge of its clinimetric properties.

Following COSMIN guidelines,^32^ this study investigated the reliability and validity (ie, test–retest reliability, measurement error, construct validity) of the IPt in post-stroke individuals. Test–retest reliability evaluates the natural within-subject variability of the performed task. Measurement error was studied to determine the amount of unit measurement needed to clinically interpret change scores beyond the measurement error. Construct validity was satisfactory as the a*-*priori hypotheses were met^32^ since IPt showed a low-to-moderate relationship with UL clinical outcomes, and distinguished between different UL severity levels.

### Reliability

The IPt was stable over the 3 evaluation sessions. The test–retest reliability was excellent (ICC >.9), and no systematic score drift was detected using the Bland–Altman method. The average number of trials performed per session was above the minimum (ie, n = 15) recommended by the SRRR for 3D functional tasks.^4^ The same researchers performed all 3 data collections at the 2 sites and received identical ~1 hour training on marker placement, system calibration, and collection procedures. These strategies helped to minimize procedural sources of error during recording.^42^ Thus, natural kinematic variability could be considered the source of the error affecting the test–retest reliability of the reaching task.^43^ Bland–Altman plots showed the presence of 2 outliers, that is, difference between measurements outside the 95% CI of the LoA_95_, for session-1 versus session-2 and session-1 versus session-3. These 2 participants had mild UL motor impairment and high UL activity scores, that is, FMA-UE: 57, ARAT: 41; FMA-UE: 65, ARAT: 57. The IPt values in one of the 3 sessions were more variable due to an increase in the effective width (We). Thus, higher variability (eg, less accuracy) at the target level influenced IPt values. Such behavior was probably due to experimental factors, rather than clinical or demographic factors. These 2 participants were not outliers regarding their clinical-demographic profiles. The same subjects showed no difference in 2 sessions. It is possible that the changed performance was due to experimental factors, or that these subjects paid less attention or did not try as hard to be accurate during all sessions. Another possibility is that fatigue may have led to more variable outcomes. However, no subject reported fatigue during or after the experimental session. Previous studies in stroke support the test–retest reliability of kinematic outcomes in similar tasks. Specifically, high ICC (ICC >.90) values were reported for the Reaching Performance Scale for Stroke, an observational kinematic assessment of compensatory movement patterns during reaching to targets located within and beyond arm workspace.^44^ Similar to our results, Patterson et al^45^ reported excellent test–retest reliability for MT (ICC = .94) and trunk displacement (ICC = .91) for a reach-to-grasp task in chronic stroke. High test–retest reliability suggests that the IPt index based on the reaching task reflected the individual’s performance at that specific time-point. No movement improvements were detected due to task repetition (ie, learning effects).

Test–retest reliability results were used to compute the error associated with the measure, that is, SEM% and MDC_95_. SEM% was less than 15%, reflecting a low measurement error. Although there are no studies to compare the IPt SEM%, our value is comparable to those reported for similar reaching tasks in post-stroke individuals. Wagner et al^38^ reported that SEM% of kinematics ranged from 2.7% to 76.8% for forward reaching tasks. Notably, similar kinematic variables used in the IPt equation showed SEM% values less than 35%, that is, MT, reach extent, and endpoint error. McCrea et al^46^ reported similar SEM% values for reaching movements (1.1%-31.6%), while in a study of UL trajectory tracking movements,^47^ SEM% values ranged from 19% to 36%. Note that all these studies constrained the trunk to intentionally prevent compensatory movement, while the present measure accounted for compensatory trunk movement.

MDC was calculated using the SEM that mathematically links measurement reliability to sample variability. The IPt showed an MDC_95_% value below 40%. Few studies reported the MDC for kinematic variables during reaching tasks in chronic stroke. In one study, Wagner et al^38^ reported MDC_95_% values between 7.4% and 98.9%. Smaller measurement errors and MDC values were described for comfortable-speed reaching. Patterson et al^45^ reported relatively small absolute MDC values for most kinematic variables, with higher absolute MDC value estimates for trunk displacement. One study in acute stroke^48^ reported that improvements in MT (5 seconds) and trunk displacement (3 cm) during a reach-to-grasp task represented clinically important changes (considering a 6pt improvement in ARAT score).

SEM and MDC_95_ are distribution-based measures that inform whether changes in IPt for each participant exceed measurement error by 14% and 39%, respectively, demonstrating statistically significant change scores. However, we did not determine the minimal amount of IPt change needed to demonstrate a clinically important difference relevant for the participant (ie, minimal clinically important difference MCID). There are no benchmarks for interpreting the SEM and MDC_95_. A MCID beyond the MDC_95_ is expected to reflect clinically important change.^49^ Further, distribution-based methods depend on sample intra-subject variability, which influences the extent of measurement error (ie, SEM is a function of SD of the observed IPt scores). Note that the IPt SD values across the 3 sessions were: ±2.62, ±2.03, and ±1.93. A probable explanation for the sample intra-subject variability lies in the distribution of study participants’ UL impairment levels.

Overall, the combination of the ICC, SEM, and MDC demonstrated excellent comprehensive reliability and suggests that the measure accurately detects change beyond measurement error. This supports the use of IPt in clinical and research settings for detecting UL sensorimotor changes in individuals with mild-to-severe stroke.

### Validity

The construct validity of the IPt was satisfactory. The IPt was developed to assess motor performance in post-stroke individuals considering the movement quality of the more-affected UL for a precision reaching task. By accounting for endpoint error, MT, and trunk displacement, the IPt is the first measure that characterizes the combination of motor performance and compensation of a skilled reaching task. The IPt showed construct validity as determined by its relationship with the FMA-UE and ARAT and discriminated between mild and moderate-to-severe stroke.

Univariate regressions investigated how much variance in FMA-UE and ARAT was explained by IPt variance. Across the 3 sessions, *R*^2^ ranged from .236 to .428 for the FMA-UE, and from 0.277 to 0.306 for the ARAT. Previous studies explored the relationships between kinematic variables and clinical measures for reaching movements in participants with various UL impairment levels. ~50% of the variance in the FMA-UE was explained by trunk displacement in pointing and reach-to-grasp tasks,^31^ but endpoint error and MT were not considered. Alt Murphy et al^50^ reported moderate correlations (−.38 < *r* < .42) between FMA-UE and kinematic variables (ie, smoothness, MT, trunk displacement). Similar to the present study, trunk displacement explained 20% of the variance in FMA-UE scores. However, the low-to-moderate relationship with the FM-UE could also be related to the fact that the IPt motor task did not involve hand movements.

On the other hand, ARAT showed a stronger relationship with kinematic variables in Alt Murphy et al^48^ than in the present study. Differences could be related to the task used (ie, grasping compared to pointing), and the number of trials (5 compared to 20). The reaching task used for computing the IPt has fewer functional-task constraints than a drinking movement (ie, no grasping component). From a motor control perspective, the nervous system organizes kinematic variables (eg, joint rotations) in an object-related and task-specific manner to achieve a movement. Therefore, the environmental affordance of the reaching task used here would be less informative than the drinking movement.^51^ However, the small number of trials used in Alt Murphy et al^50^ reduced the estimation of the natural inter-subject kinematic variability used for the task.^43^ Finally, in the present study, participants had a greater range of sensorimotor impairment. Recently, Rech et al^52^ found a high correlation between UL kinematics and FMA-UE. The higher correlation compared to the present one may be due to the closer location of the target to the subject’s body (ie, 80% arm-length) and the performance of the movement limited to the horizontal plane. However, clinical measures do not specifically require precision reaching skills and are therefore not expected to correlate highly with such a focused measure. Indeed, our finding of a low-to-moderate relationship between IPt and clinical UL motor impairment and activity scores suggest that the IPt as a valid measure that can provide information about motor skill acquisition distinct from clinical assessments.

ROC analyses suggests that IPt distinguishes between levels of sensorimotor impairment severity based on the FMA-UE score (mild: ≥50/66 and moderate-to-severe: ≤49/66). In accordance with the present results, earlier studies reported the discriminative ability of individual and multi-kinematic variables to discriminate between different levels of UL impairment in late sub-acute and chronic stroke. The IPt accounts for sagittal trunk displacement that is considered a major compensatory movement during reaching tasks.^30^ Furthermore, evidence suggests that trunk displacement alone discriminates between levels of motor impairment.^31^ Recently, observational kinematic measures of endpoint movement, sagittal trunk displacement, shoulder, and elbow movement used to quantify movement patterns during forward-reaching, demonstrated similar discriminative validity characteristics.^44^

ROCs provided sensitivity (50%-83.33%) and specificity (81%-100%) characteristics of IPt with respect to different UL impairment levels. Thus, IPt has a high probability of correctly classifying individuals with mild UL impairment, that is, correctly classifying an individual as testing negative when disease is absent (considering mild impairment *disease-free*). Therefore, the false positive rate is lower than the false-negative rate. This suggests that IPt can accurately identify people who do not have moderate-to-severe hemiparesis. The ability of IPt to more accurately identify individuals with different severity levels than simple trunk displacement, or single UL kinematic measures may provide essential information to tailor rehabilitation interventions and potentially enhance patient outcomes. The IPt frames endpoint performance (accuracy, speed) and movement quality (trunk displacement) with a single value, and may provide a valid and efficient metric of motor skills for identifying stroke severity. IPt is also an interval-scaled measure that overcomes the limitations of ordinal measures such as lack of responsiveness.^53^ Thus, the IPt characterizing essential motor skills may contribute to artificial intelligence approaches such as machine-learning or decision tree-based classification algorithms for single-subject predictions in rehabilitation programs and research.^54^

### Limitations

The generalizability of IPt results is limited to different levels of stroke chronicity (>3 months post-stroke) and to the specific reaching task performed. Future studies should evaluate IPt reliability and validity for other UL tasks in acute stroke and different patient populations. The IPt was computed using kinematic data collected with a highly-precise motion capture system. Therefore, its clinimetric properties when derived from low-cost inertial measurement units needs further investigation.

## Conclusions

The novel IPt for a reaching task incorporates performance and movement quality variables and is a promising measure for research and clinical settings. This study provides evidence for the reliability and validity of IPt for a reaching task. IPt may be used in conjunction with other impairment and activity outcome scales to further describe the motor profile and motor skill acquisition in post-stroke individuals. Future studies should investigate the predictive power and the minimal clinically significant difference of IPt in stroke survivors.

## References

[1] Kwakkel G Lannin NA Borschmann K English C , et al. Standardized measurement of sensorimotor recovery in stroke trials: consensus-based core recommendations from the Stroke Recovery and Rehabilitation Roundtable. Int J Stroke. 2017;12:451-461. 28697709 10.1177/1747493017711813

[2] Zeiler SR Krakauer JW. The interaction between training and plasticity in the poststroke brain. Curr Opin Neurol. 2013;26:609-616. 24136129 10.1097/WCO.0000000000000025 PMC4012223

[3] Saes M Mohamed Refai MI van Beijnum BJF , et al. Quantifying quality of reaching movements longitudinally post-stroke: a systematic review. Neurorehabil Neural Repair. 2022;36(3):183-207. doi:10.1177/15459683211062890 35100897 PMC8902693

[4] Kwakkel G Van Wegen EEH Burridge JH , et al. Standardized measurement of quality of upper limb movement after stroke: consensus-based core recommendations from the Second Stroke Recovery and Rehabilitation Roundtable. Neurorehabil Neural Repair. 2019;33(11):951-958. doi:10.1177/1545968319886477 31660781

[5] Shmuelof L Krakauer JW Mazzoni P. How is a motor skill learned? Change and invariance at the levels of task success and trajectory control. J Neurophysiol. 2012;108(2):578-594. 22514286 10.1152/jn.00856.2011 PMC3404800

[6] Fitts PM. The information capacity of the human motor system in controlling the amplitude of movement. J Exp Psychol. 1954;7(6):381-391. 13174710

[7] Michaelsen SM Jacobs S Roby-Brami A Levin MF. Compensation for distal impairments of grasping in adults with hemiparesis. Exp Brain Res. 2004;157:162-173. doi:10.1007/s00221-004-1829-x 14985899

[8] Michaelsen SM Luta A Roby-Brami A Levin MF. Effect of trunk restraint on the recovery of reaching movements in hemiparetic patients. Stroke. 2001;32:1875-1883. 11486120 10.1161/01.str.32.8.1875

[9] Hseih TY Pacheco MM Liu YT Newell KM. Are sub-movements induced visually in discrete aiming tasks? J Mot Behav. 2021;17:1-13. doi:10.1080/00222895.2021.1937031 34139963

[10] Kelso JA Southard DL Goodman D. On the nature of human interlimb coordination. Science. 1979;203(4384):1029-1031. doi:10.1126/science.424729 424729

[11] Schwarz A Kanzler CM Lambercy O Luft AR Veerbeek JM. Systematic review on kinematic assessments of upper limb movements after stroke. Stroke. 2019;50:718-727. doi:10.1161/STROKEAHA.118.023531 30776997

[12] Smits-Engelsman BCM Wilson PH Westenberg Y Duysens J . Fine motor deficiencies in children with developmental coordination disorder and learning disabilities: an underlying open-loop control deficit. Hum Mov Sci. 2003;22 (4-5):495-513. doi:10.1016/j.humov.2003.09.006 14624830

[13] Hammerbeck U Yousif N Hoad D Greenwood R Diedrichsen J Rothwell JC. Chronic stroke survivors improve reaching accuracy by reducing movement variability at the trained movement speed. Neurorehabil Neural Repair. 2017;31:499-598. doi:10.1177/1545968317693112 28506150

[14] Sanes JN Dimitrov B Hallett M. Motor learning in patients with cerebellar dysfunction. Brain. 1990;113(Pt 1):103-120. doi:10.1093/brain/113.1.103 2302528

[15] Alouche S Banina M Lam TK , et al. Using biomarkers of neuroimaging and kinematic measures to explain the variability in stroke motor impairment. American Society of Neurorehabilitation Annual Meeting; November 2019; Chicago, IL.

[16] Gowland C Stratford P Ward M , et al. Measuring physical impairment and disability with the Chedoke-McMaster Stroke Assessment. Stroke. 1993;24(1):58-63. 8418551 10.1161/01.str.24.1.58

[17] Gowland C Van Hullenaar S Torresin W. Chedoke-McMaster Stroke Assessment: Development, Validation and Administration Manual; 1995.

[18] Bohannon RW Smith MB. Interrater reliability of a modified Ashworth scale of muscle spasticity. Phys Ther. 1987;67(2):206-207. 3809245 10.1093/ptj/67.2.206

[19] Nasreddine ZS Phillips NA Bédirian V , et al. The Montreal Cognitive Assessment, MoCA: a brief screening tool for mild cognitive impairment. J Am Geriatr Soc. 2005;53(4):695-699. 15817019 10.1111/j.1532-5415.2005.53221.x

[20] Dong Y Sharma VK Chan BP-L , et al. The Montreal Cognitive Assessment (MoCA) is superior to the Mini-Mental State Examination (MMSE) for the detection of vascular cognitive impairment after acute stroke. J Neurol Sci. 2010;299(1-2):15-18. 20889166 10.1016/j.jns.2010.08.051

[21] Stamenova V Roy EA Black SE. Associations and dissociations of transitive and intransitive gestures in left and right hemisphere stroke patients. Brain Cogn. 2010;72(3):483-490. 20167414 10.1016/j.bandc.2010.01.004

[22] Stamenova V Black SE Roy EA. An update on the Conceptual-Production Systems model of apraxia: evidence from stroke. Brain Cogn. 2012;80(1):53-63. 22634032 10.1016/j.bandc.2012.03.009

[23] Leibovitch FS Vasquez BP Ebert PL Beresford KL Black SE. A short bedside battery for visuoconstructive hemispatial neglect: Sunnybrook Neglect Assessment Procedure (SNAP). J Clin Exp Neuropsychol. 2012;34(4):359-368. 22260299 10.1080/13803395.2011.645016

[24] Oldfield R. The assessment and analysis of handedness: the Edinburgh inventory. Neuropsychologia. 1971;9:97-113. 5146491 10.1016/0028-3932(71)90067-4

[25] Fugl-Meyer AR Jaasko L Leyman I Olsson S Steglind S . The post-stroke hemiplegic patient. 1. A method for evaluation of physical performance. Scand J Rehabil Med. 1975;7:13-31. 1135616

[26] Gladstone DJ Danells CJ Black SE. The Fugl-Meyer assessment of motor recovery after stroke: a critical review of its measurement properties. Neurorehabil Neural Repair. 2002;16(3):232-240. 12234086 10.1177/154596802401105171

[27] Platz T Pinkowski C van Wijck F Kim IH di Bella P Johnson G. Reliability and validity of arm function assessment with standardized guidelines for the Fugl-Meyer Test, Action Research Arm Test and Box and Block Test: a multicentre study. Clin Rehabil. 2005;19(4):404-411. 15929509 10.1191/0269215505cr832oa

[28] Yozbatiran N Der-Yeghiaian L Cramer SC. A standardized approach to performing the action research arm test. Neurorehabil Neural Repair. 2008;22(1):78-90. 17704352 10.1177/1545968307305353

[29] Howard IS Ingram JN Körding KP Wolpert DM. Statistics of natural movements are reflected in motor errors. J Neurophysiol. 2009;102(3):1902-1910. doi:10.1152/jn.00013.2009 19605616 PMC2746767

[30] Cirstea MC Levin MF. Compensatory strategies for reaching in stroke. Brain. 2000;123:940-953. 10775539 10.1093/brain/123.5.940

[31] Subramanian SK Yamanaka J Chilingaryan G Levin MF. Validity of movement pattern kinematics as measures of arm motor impairment poststroke. Stroke. 2010;41(10):2303-2308. 20814001 10.1161/STROKEAHA.110.593368

[32] Mokkink LB Terwee CB Patrick DL , et al. The COSMIN study reached international consensus on taxonomy, terminology, and definitions of measurement properties for health-related patient-reported outcomes. J Clin Epidemiol. 2010;63:737-745. 20494804 10.1016/j.jclinepi.2010.02.006

[33] Rosner B Donner A Hennekens CH. Significance testing of interclass correlations from familial data. Biometrics. 1979;35:461-471. doi:10.2307/2530348

[34] Portney LG Watkins MP. Foundations of Clinical Research: Applications to Practice. 3rd ed. FA Davis; 2015.

[35] Bland JM Altman DG. Statistical methods for assessing agreement between two methods of clinical measurement. Lancet. 1986;1:307-310. 2868172

[36] de Vet HC Terwee CB Mokkink L Knol DL. Measurement in Medicine. Cambridge University Press; 2011.

[37] Weir JP. Quantifying test-retest reliability using the intraclass correlation coefficient and the SEM. J Strength Cond Res. 2005;19:231-240. 15705040 10.1519/15184.1

[38] Wagner JM Rhodes JA Patten C. Reproducibility and minimal detectable change of three-dimensional kinematic analysis of reaching tasks in people with hemiparesis after stroke. Phys Ther. 2008;88(5):652-663. doi:10.2522/ptj.20070255 18326055

[39] Duncan PW Goldstein LB Horner RD Landsman PB Samsa GP Matchar DB. Similar motor recovery of upper and lower extremities after stroke. Stroke. 1994;25:1181-1188. 8202977 10.1161/01.str.25.6.1181

[40] Prinsen CA Vohra S Rose MR , et al. How to select outcome measurement instruments for outcomes included in a “Core Outcome Set”–a practical guideline. Trials. 2016;17:449. 27618914 10.1186/s13063-016-1555-2 PMC5020549

[41] Levin MF Kleim JA Wolf SL. What do motor “recovery” and “compensation” mean in patients following stroke? Neurorehabil Neural Repair. 2009;23(4):313-319. 19118128 10.1177/1545968308328727

[42] McGinley JL Baker R Wolfe R Morris ME. The reliability of three-dimensional kinematic gait measurements: a systematic review. Gait Posture. 2009;29(3):360-369. doi:10.1016/j.gaitpost.2008.09.003 19013070

[43] Bernstein NA. The Co-ordination and Regulation of Movements. Pergamon Press; 1967.

[44] Subramanian SK Baniña MC Turolla A Levin MF. Reaching performance scale for stroke - Test-retest reliability, measurement error, concurrent and discriminant validity. PM R. 2022;14(3):337-347. doi:10.1002/pmrj.12584 33675151

[45] Patterson TS Bishop MD McGuirk TE Sethi A Richards LG. Reliability of upper extremity kinematics while performing different tasks in individuals with stroke. J Mot Behav. 2011;43:121-130. 21347950 10.1080/00222895.2010.548422

[46] McCrea PH Eng JJ Hodgson AJ. Saturated muscle activation contributes to compensatory reaching strategies after stroke. J Neurophysiol. 2005;94(5):2999-3008. doi:10.1152/jn.00732.2004 16014786 PMC3471982

[47] Patten C Kothari D Whitney J Lexell J Lum PS. Reliability and responsiveness of elbow trajectory tracking in chronic poststroke hemiparesis. J Rehabil Res Dev. 2003;40:487-500. 15077661 10.1682/jrrd.2003.11.0487

[48] Alt Murphy M Willén C Sunnerhagen KS . Responsiveness of upper extremity kinematic measures and clinical improvement during the first three months after stroke. Neurorehabil Neural Repair. 2013;27(9):844-853. doi:10.1177/1545968313491008 23764883

[49] Prinsen CA Mokkink LB Bouter LM , et al. COSMIN guideline for systematic reviews of patient-reported outcome measures. Qual Life Res. 2018;27(5):1147-1157. doi:10.1007/s11136-018-1798-3 29435801 PMC5891568

[50] Alt Murphy M Willén C Sunnerhagen KS . Movement kinematics during a drinking task are associated with the activity capacity level after stroke. Neurorehabil Neural Repair. 2012;26(9):1106-1115. doi:10.1177/1545968312448234 22647879

[51] Gibson JJ. The Ecological Approach to Visual Perception. Classic ed. Psychology Press; 2015.

[52] Rech KD Salazar AP Marchese RR Schifino G Cimolin V Pagnussat AS. Fugl-Meyer assessment scores are related with kinematic measures in people with chronic hemiparesis after stroke. J Stroke Cerebrovasc Dis. 2020;29(1):104463. doi:10.1016/j.jstrokecerebrovasdis.2019.104463 31740027

[53] Piscitelli D Pellicciari L. Responsiveness: is it time to move beyond ordinal scores and approach interval measurements? Clin Rehabil. 2018;32(10):1426-1427. doi:10.1177/0269215518794069 30114940

[54] Bonkhoff AK Grefkes C. Precision medicine in stroke: towards personalized outcome predictions using artificial intelligence. Brain. 2022;145:457-475. doi:10.1093/brain/awab439 34918041 PMC9014757

